# Graphene-Based Chemiresistor Sensors for Drinking Water Quality Monitoring

**DOI:** 10.3390/s23249828

**Published:** 2023-12-14

**Authors:** Mason McGarrity, Feng Zhao

**Affiliations:** Micro/Nanoelectronic and Energy Laboratory, School of Engineering and Computer Science, Washington State University, Vancouver, WA 98686, USA; mason.mcgarrity@wsu.edu

**Keywords:** chemiresistor, graphene, graphene-oxide, reduced graphene-oxide, sensor, water quality, sensitivity

## Abstract

Monitoring the quality of drinking water is a crucial responsibility for all water infrastructure networks, as it guarantees access to clean water for the communities they serve. With water infrastructure deteriorating due to age and neglect, drinking water violations are on the rise in the US, underscoring the need for improved monitoring capabilities. Among the different sensor technologies, graphene-based chemiresistors have emerged as a promising technology for water quality monitoring due to advantages such as simple design, sensitivity, and selectivity. This review paper provides an overview of recent advances in the development of graphene-based chemiresistors for water quality monitoring, including principles of chemiresistive sensing, sensor design and functionalization, and performance of devices reported in the literature. The paper also discusses challenges and opportunities in the field and highlights future research directions. The development of graphene-based chemiresistors has the potential to revolutionize water quality monitoring by providing highly sensitive and cost-effective sensors that can be integrated into existing infrastructure for real-time monitoring.

## 1. Introduction

The quality of drinking water is fundamental to overall human health and well-being. Despite increased awareness and emphasis on environmental health and safety, water quality monitoring (WQM) remains a chronically underdeveloped area with respect to technological advancement. Reliable water infrastructure is fundamental in ensuring that communities receive clean and safe water. Despite its instrumental role, water infrastructure in the U.S. has been overlooked and is experiencing decline in various regions, with the renewal and replacement of aging water infrastructure ranked by water utilities as the most pressing issue facing the water industry in 2022 [1]. The deterioration of infrastructure, caused by a combination of aging and neglect, is further exacerbated by pressing challenges such as increased demand, pollution, and limited funding [1]. All these factors contribute to public water systems (PWSs) being non-compliant with regulations set by the United States Environmental Protection Agency (EPA). Each year, the EPA publishes a report on the drinking water violations committed by PWSs. The most recent publicly available report is from 2021 and it states that 25% (38,853) of PWSs were reported to have violated a least 1 drinking water standard, with approximately 19% (28,828) being monitoring or reporting related [2]. The current state of water infrastructure in the U.S. is characterized by a patchwork of water networks, expanded to meet the needs of rapidly growing communities. This rapid growth has inevitably resulted in large and convoluted systems, with each subsection constructed using the materials and methods that were considered the industry standard at that time. This leaves water authorities with the challenging responsibility of maintaining these systems, as well as identifying and replacing sections that were constructed with materials that are now known to be hazardous, such as lead [3]. To better equip the water authorities with these monitoring tasks, the development of effective and affordable sensors is crucial. In this context, graphene-based chemiresistors are promising candidates. Both the unique material properties of graphene and the economical and ease-of-use advantages offered by chemiresistive topology make them ideal for use in the water industry.

Regarding U.S. water quality regulations, the Safe Drinking Water Act (SDWA) is a cornerstone piece of legislation. Enacted in 1974 and continuously amended, the SDWA, a federal law administered by the Environmental Protection Agency (EPA), is a critical framework for safeguarding public health [4]. By establishing and enforcing national standards for drinking water quality, the SDWA regulates contaminants and treatment techniques, ensuring that public water systems adhere to stringent guidelines. Compliance with these standards is mandatory, underscored by the Act’s emphasis on transparency. The SDWA requires water systems to provide consumers with annual water quality reports, enhancing public awareness and engagement. This regulatory foundation, coupled with the EPA’s periodic updates of regulatory limits and treatment techniques, forms a comprehensive approach. Public water systems are mandated to regularly monitor their water quality and submit reports to the EPA and state regulatory agencies. In cases of violations, the EPA can take action, reinforcing the commitment to upholding the quality and safety of drinking water [5].

One of the challenges facing the establishment of a robust network of water quality sensors is the fact that contaminants can be introduced at any point in the infrastructure system, and probability calculation to determine the likelihood of contamination occurring at a given point is nontrivial [6]. Failures in transmission and distribution mains can interrupt the delivery of water and introduce contaminants from the surrounding environment into the drinking water supply after it passes through the water treatment plant. Water main breakages and subsequent contamination represent a real threat, substantiated by data showing that between 250,000 and 300,000 water main breaks occur every year in the U.S. [7]. Another source of contamination is from the mains being constructed from materials toxic to human health, such as lead, or materials that corrode over time, deteriorating water quality [8]. For example, the results of a national survey of community water systems conducted by the American Water Works Association (AWWA), found that approximately 15 to 22 million people in the U.S. receive their drinking water from networks consisting of full or partial lead service lines [9]. It is not uncommon for a water utility to be unaware of a lead service line in their transmission and distribution network until a break occurs and the lead service line is exposed. The same report from the AWWA found that a portion of community water system operators responded “Not Sure” when asked to report the presence of lead service lines within their water network. A third method of drinking water supply contamination comes from events external to the water system, resulting in the release of foreign material into the surrounding environment. This material can then penetrate the local drinking water supply through various means, such as contamination of surface or groundwater. One such event was the environmental disaster stemming from the derailment of railcars in Ohio that were carrying substantial quantities of the volatile organic compound (VOC) vinyl chloride, a known human carcinogen [10]. This spill and the subsequent controlled burn of the substance resulted in significant contamination of the local environment (of both air and water). It was reported that an estimated 7.5 miles of surface waterway had been contaminated [11]. The need for affordable, scalable, and continuous methods of WQM is paramount to ensure that all communities have confidence in their drinking water quality.

Other challenges in effective water quality monitoring that are inherent to the sensors themselves include both the number of contaminants as well as the complex real-world environment in which the sensors must detect [12]. In the United States, the list of contaminants is maintained by the EPA, a government agency in charge of determining safe levels for chemicals and other pollutants in food and water, among other environmentally orientated goals. At the time of writing, eighty-nine contaminants are listed in the “National Primary Drinking Water Regulations” [13], with some representative examples listed in Table 1. Each contaminant on this list presents a unique design challenge in developing a sensor with the necessary sensitivity to respond to the target analyte at the necessary concentration level that indicates if the water sample passes regulation limits. Additionally, it is critical that a sensor not only detect the analyte in a simple lab sample but also in a complex real-world sample consisting of the target intermixed in a highly variable solution. In other words, to be effective, a sensor must exhibit a high degree of both sensitivity and selectivity.

### 1.1. Nanotechnology for Water Quality Monitoring

The current industry standard methods of WQM involve the use of lab-based instrumentation to evaluate the water sample collected at the source of interest. This method is expensive, labor intensive, and low-throughput, and while the sensitivity of the laboratory methods is high, it is only possible to gain a low-resolution view of the water system from a temporal perspective due to the limited periodicity of the sampling [14]. The widespread deployment of inexpensive nanosensor devices would enable water authorities’ access to continuous real-time data, providing—for the first time—a high-resolution, comprehensive view of the water quality in their systems [14,15]. A nanotechnology-based approach offers several advantages over traditional water quality monitoring methods, beyond just the ability to provide continuous and real-time data. For example, because nanosensors can be integrated into small, low-power devices, they offer the potential for the on-site monitoring and transmission of data in remote or hard-to-reach areas [16]. Additionally, because nanosensors can be designed to be highly specific to certain analytes, they can be used to detect and monitor a wide range of contaminants and pollutants, including heavy metals, organic compounds, and pathogens [12,16,17,18]. Finally, the use of nanosensors in water quality monitoring has the potential to reduce the overall cost of monitoring, as they are cheaper to manufacture than benchtop equipment and often do not require trained personnel to operate or maintain [18].

While many viable types and topologies of nanosensors exist [18], this review aims to provide a targeted and concise presentation of a group of nanosensors referred to as “chemiresistors”. More specifically, this review will consider chemiresistive devices that utilize graphene or graphene derivatives and are designed to sense analytes relevant to drinking water quality in an aqueous environment. The sensors reviewed in this paper meet the following criteria: the devices are operated based on a potentiometric principle; the materials used for the sensors are graphene or a graphene derivative; and the detection of analytes is performed in an aqueous environment. The relevant sensor designs are grouped by target analyte classification, following the same methodology as the EPA’s National Primary Drinking Water Regulations in Table 1.

### 1.2. Chemiresistors

**Figure 1:**
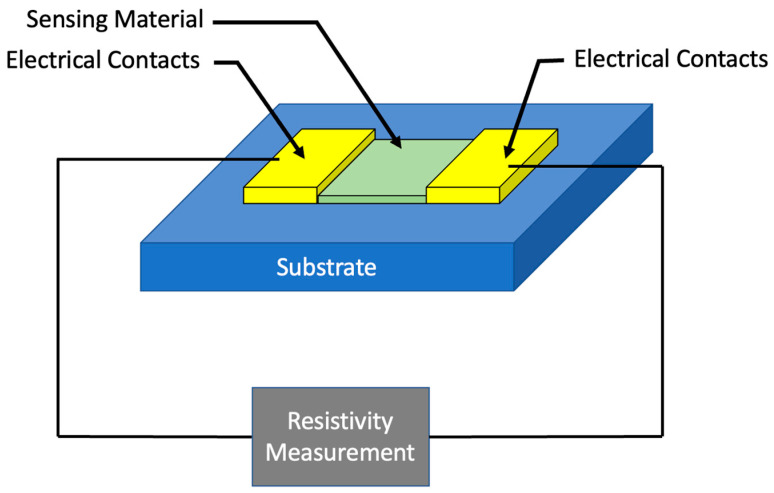
Architecture of a basic chemiresistor.

A chemiresistor (a conjunction of “chemical” and “resistor”) is a type of sensor whose electrical resistivity changes as a result of interactions with the surrounding environment. The architecture of a basic chemiresistor is shown in Figure 1. A chemiresistor can be designed to respond to a wide variety of target analytes, depending on the choice of sensing material and/or functionalization of that material [19]. The resistance of the device is calibrated in an environment with no analyte present. Once calibrated, the sensor can be exposed to varying concentrations of the analyte and the subsequent change in device resistance during each exposure can be measured to characterize the device and extract critical figures of merit (FoM), such as sensitivity and limit of detection (LoD). Chemiresistors are solid-state devices with a simple structure, constructed from two electrical contacts connected by some material (in this review, graphene) to serve as the sensor’s interface with the surrounding environment. To improve device selectivity, this sensing surface will often be functionalized in such a way as to increase the proclivity of the surface to bind with the target analyte and reject other interacting species that would serve to “confuse” the sensor and take up valuable interaction surface [20]. While the fundamental device structure of a chemiresistor is not complex, device performance is strongly dependent on the sensor geometry, choice of sensing material, and functionalization techniques. For sensors used in the context of WQM, precision is paramount as often the regulatory limit for analytes is very low; therefore, the material used as the sensing interface must be highly sensitive to the external environment. Graphene is a material that serves this purpose well and has been heavily used in other types of sensors, such as those for various gasses [21,22]. Additionally, the contacts are typically physically and electrically isolated from the environment to reduce the amount of noise impacting the performance of the device.

### 1.3. Graphene and Its Derivatives

Graphene, a two-dimensional carbon allotrope with a honeycomb-shaped lattice, has been a focal point of intensive research since its isolation from bulk graphite through micromechanical cleavage in 2004 [23]. With an outstanding electrical conductivity marked by carrier mobility exceeding 15,000 cm²/Vs, graphene proves to be an ideal material for efficient charge transport in electronic devices. Additionally, its extraordinary thermal conductivity of over 4000 W/mK surpasses traditional materials, positioning graphene as a promising candidate for applications in heat dissipation and thermal management. The material’s remarkable mechanical strength, boasting a fracture strength of approximately 130 GPa, makes it one of the strongest known materials, suitable for applications requiring robust structural integrity [24,25,26]. Graphene’s flexibility, transparency, and biocompatibility further enhance its utility, allowing it to conform to different surfaces, making it particularly valuable in flexible electronics, and enabling its use in transparent conductive films and optoelectronic devices. These specific properties collectively contribute to graphene’s versatility, driving advancements in electronics, materials science, and various cutting-edge technologies. This has spurred the development of various graphene-based sensors, including electrochemical sensors, strain sensors, electrical sensors, and flexible sensors [27,28,29,30,31]. For an in-depth exploration of the material properties of graphene, additional details can be found elsewhere [24,32].

While the development of various techniques to synthesize graphene is an active area of research, some of the notable methods of bulk graphene production include chemical vapor deposition (CVD), mechanical exfoliation, and the improved Hummer’s method [27,33,34,35,36]. Of the three methods of graphene synthesis listed above, broadly speaking, CVD produces the highest-quality film due to its high controllability [33], mechanical exfoliation offers a low-cost approach [34], and the improved Hummer’s method is an efficient way to produce graphene-oxide (GO) [35]. After the graphene has been synthesized, the resulting product can be binned into the following categories based on the number of graphene sheets in the stack: single or monolayer graphene (SLG), few-layer graphene (FLG) (2–5 sheets), and multilayer graphene (MLG) (6–10 sheets).

**Figure 2:**
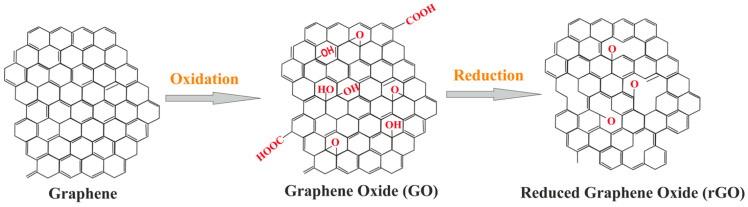
Conversion of graphene into its derivatives [42].

Two of the most prevalent graphene derivatives are graphene-oxide (GO) and reduced graphene-oxide (rGO). A schematic conversion of graphene into its derivatives is shown in Figure 2. Unlike pristine graphene, GO is not a recent discovery, having first been synthesized from graphite in 1859 [37]. While GO maintains a 2D carbon structure, it has additional oxygen-containing functional groups attached on both the sides and edges of the carbon plane, resulting in severely diminished material properties [36] when compared to pristine graphene. The major advantage offered by GO is its relative ease of bulk synthesis from graphite through well-documented methods, such as Hummer’s or improved Hummer’s [36,37]. To better emulate the characteristics of pristine graphene, GO is commonly reduced via thermal or chemical methods to strip away the excess functional groups, resulting in rGO [38]. Depending on the requirements of the intended application, rGO can offer a satisfactory compromise between the difficulty or cost of obtaining high-quality monolayer graphene and the diminished material properties of GO.

Through interactions with the surrounding environment, graphene’s characteristics can be modified. Two crucial methods of interaction include ion–π interactions and π-π interactions. In graphene, π-π interactions refer to non-covalent interactions between the π electrons of adjacent aromatic systems. Graphene, composed of a hexagonal lattice of carbon atoms, features delocalized π electrons forming π bonds above and below the plane of carbon atoms [39]. The significance of π-π interactions in graphene arises from its extended, planar, and aromatic structure. Each carbon atom in a graphene sheet is sp^2^ hybridized, allowing π electrons to move freely along the entire conjugated system. When graphene sheets come close to other aromatic systems, such as another graphene sheet, their π electron clouds interact through attractive van der Waals forces, specifically via π-π stacking. This interaction allows graphene surfaces to be readily functionalized with molecules that have a specific affinity for a target analyte. Furthermore, π-π stacking can be leveraged to induce a direct interaction between graphene and other aromatic target analytes, altering the graphene layer’s properties, such as electrical conductivity, through the adsorption of aromatic compounds [40,41].

Ion-π interactions involve attractive forces between positively charged ions (cations) and the negatively charged π electron cloud of graphene. Cations are attracted to the electron-rich regions above and below the graphene plane through electrostatic forces. This interaction results in the adsorption of ions onto the graphene surface, influencing the electrical properties of both the ions and the graphene. This adsorption phenomenon modifies the charge distribution on the graphene surface, playing a significant role in modulating the electrical conductivity of graphene [40,41].

## 2. Graphene-Based Chemiresistor

When evaluating a sensor design, three primary FoM regarding device performance need consideration: sensitivity, selectivity, and response time. Additionally, other factors like the ability to reset the sensor multiple times, device lifespan, and manufacturing cost are also important to consider. However, if a sensor cannot meet the necessary requirements for sensitivity, selectivity, and response time for a target analyte, these secondary factors become irrelevant. The sensitivity of a sensor refers to its ability to detect and quantify small changes in the concentration of the target analyte. A highly sensitive sensor will require a lower concentration of the target analyte to interact with the device in order to produce a measurable response; thus, a device with a higher sensitivity will be able to detect lower concentrations of the target analyte. Selectivity, on the other hand, refers to the sensor’s ability to distinguish the target analyte from other interfering substances present in the sample. A highly selective sensor will respond only to the target analyte, minimizing false positives and false negatives. The response time is the time required for the sensor to reach its stable output value after being exposed to the target analyte. A faster response time allows for more rapid detection and analysis of the target analyte. At very low concentrations, the response time can become a constraining factor as the concentration of the target analyte in the sample is extremely low and it must reach the interaction surface of the sensor within a reasonable time. Therefore, all three characteristics are important and should be optimized to achieve the best sensor performance [43,44]. A fourth FoM that is often reported for sensors is the LoD. This value represents the lowest concentration of an analyte that can be reliably distinguished from a blank sample containing no analytes, i.e., the smallest signal distinguishable from the noise [45]. This value is calculated from experimentally obtained data and is often significantly lower than the lower limit of the measured values. While there are different methods of calculating the LoD [46], the values reported in this review are as given and are only provided where explicitly reported in the corresponding reference.

Graphene-based chemiresistors stand out as a leading choice among sensors due to several key advantages. Regarding the choice of sensing material, graphene stands out when compared to other popular nanomaterial choices, such as carbon nanotubes or silicon nanowires. As discussed in Section 1.3, graphene offers remarkable carrier mobility, carrier density, and low intrinsic noise. The physical properties of graphene, such as its two-dimensional (2D) structure, allow for all atoms to be exposed to the surrounding environment, creating an extremely large surface-to-volume ratio and a maximum number of locations for interaction with the analyte [47,48,49]. This 2D structure also allows for greater ease of surface functionalization and since graphene is an aromatic compound [50] it readily interacts with other molecules containing aromatic rings, giving graphene enhanced versatility when it comes to detecting a broader range of analytes.

A thorough understanding of the relevant sensing mechanisms is critical. In a graphene-based chemiresistor, the electrical double layer (EDL) at the graphene-electrolyte interface plays a crucial role in influencing the device’s performance [51]. The formation of the EDL involves the adsorption of ions from the electrolyte solution onto the graphene surface, creating an inner Helmholtz plane (IHP) of charged particles [52,53]. This structure has a pronounced impact on the charge distribution and electronic properties of graphene. Importantly, the electrostatic gating effect, closely associated with the EDL, allows for precise modulation of the charge carrier concentration in graphene by applying an external voltage [54]. In a chemiresistor, this phenomenon is instrumental in detecting analytes, as changes in the EDL induced by the presence of specific molecules lead to alterations in graphene’s electrical conductivity.

Another way the electrical characteristics of graphene can be modified is through a phenomenon known as swelling. Swelling in graphene arises from various mechanisms, primarily involving the adsorption of molecules or ions onto its surface, inducing interlayer separation [55]. The presence of functional groups on graphene surfaces facilitates interactions, contributing to swelling. Importantly, these swelling mechanisms have a profound impact on graphene’s electrical characteristics. The tuning of interlayer spacing influences charge carrier concentration and mobility, making swollen graphene a versatile material for sensors. Controlled swelling can be harnessed in sensor design, where the adsorption-induced changes in electrical conductivity serve as a sensitive indicator for the presence and concentration of specific analytes, highlighting the significance of understanding swelling mechanisms in tailoring graphene properties for sensor applications.

The chemiresistor architecture presents distinct advantages compared to alternative microelectronic sensor architectures, such as field-effect transistors (FETs). Its simplicity in design is a key attribute, enabling a faster, more reproducible, and cost-effective fabrication process. The inherent reliability of the chemiresistor design further enhances its attractiveness, as it minimizes the potential points of failure and increases overall sensor robustness. When paired with graphene, these chemiresistive sensors are an appealing choice in fields where high precision, cost-effectiveness, and reliable sensing are paramount.

Table 2 summarizes the representative graphene-based chemiresistor designs that are reviewed in greater detail in the following sub-sections.

### 2.1. pH Detection

pH is a measurement of how acidic or basic a solution is. The method of determining the pH of a solution is through the application of a negative logarithm to the concentration of hydrogen ions measured in the sample under test (Equation (1)).

(1) $$pH=-\log_{10}\left[H_{3}O^{+}\right]$$

While not a direct measurement of any contaminants, the pH of water is an important metric for assessing water quality and is measured on a scale ranging from 1 (acidic) to 14 (basic), with a pH of 7 being neutral. Two areas of water quality that are heavily influenced by pH levels are disinfection and corrosion control [72]. Disinfection refers to the addition of chemicals into the water supply to combat the growth of microorganisms (Section 2.2). One example of how pH plays a critical role in the effectiveness of water disinfection is in the use of chlorine, a well-known disinfectant. When chlorine is introduced into water, it quickly undergoes a chemical reaction to produce hydrochloric acid (HCl) and hypochlorous acid (HOCl) [73]. HOCl is a weak acid that undergoes partial dissociation into a hydrogen ion (H^+^) and a hypochlorite ion (OCl^−^); however, when regarding disinfection, HOCl is the primary actor. As described by Equation (1), the pH is expressed as the concentration of hydrogen ions; therefore, the overall pH of the water containing chlorine will have a significant impact on the disassociation reaction and final concentration of HOCl available for disinfection. Higher, more basic, pH levels result in a lower concentration of HOCl, while lower, more acidic levels result in a higher concentration. To underline the importance of pH on the concentration of HOCl, it has been shown that in an environment with a pH of 6, the percentage of free chlorine in the HOCl form is approximately 96.8%. However, when the pH was increased to 8, the amount of free chlorine in the HOCl form was only roughly 23.3% [74]. The interaction between these disinfectants and naturally occurring organic matter in the water supply produces disinfection byproducts (DBPs) [75]. While the formation of DBPs is dependent on many variables, it is known that pH plays a role in modulating the concentrations of these chemicals [76,77].

Additionally, monitoring pH in a water system is important for controlling corrosion within the system [78]. Corrosion is a complex process that is influenced by several factors, and pH is a key determinant. Deviations from the optimal pH for the materials in the system can accelerate the corrosion, leading to the release of heavy metals into the water, posing health risks and compromising the infrastructure integrity [79]. For example, one study [80] examining the release of manganese from pipes in a drinking water distribution system found a statistically significant difference in the amount of manganese released under three different pH conditions, with lower pH samples releasing greater amounts of manganese than higher pH samples.

At present, regulations have not been established by the EPA or World Health Organization (WHO) for pH values of drinking water. However, the EPA recommends that the pH of drinking water be within a range of 6.5 to 8.5 [81]. Regular monitoring of pH levels is critical for allowing water authorities to promptly detect and address any deviations, safeguarding the public from potential health hazards and ensuring the overall reliability and sustainability of the drinking water supply [72].

So far, four graphene-based chemiresistive devices have been reported in the literature, where three of the devices [56,57,58] relied on the direct interaction between the analyte and the graphene sensing surface, while the fourth device [59] leveraged the functionalization of the interaction surface with various pyrene derivatives. Each reported device employed a different form of graphene and different material composition for the electrical contacts. Key performance metrics of the pH sensors reviewed can be found in Table 3.

Early work in the area of graphene-based chemiresistor sensors [56] employed the simple method of mechanical exfoliation of graphite to produce the graphene used in the device. Another direct interaction device was reported by [57], leveraging dielectrophoresis during the deposition of graphene sheets. This technique enables a more targeted approach to the final location of the graphene sheets, which reduces the random dispersion and, therefore, device-to-device variation. The device reported in [58] differs from the other two devices in that a vacuum filtration technique was utilized to deposit the graphene onto a paper substrate. Direct interaction designs [56,57] demonstrated repeatability between measurements as well as similar effective pH ranges, while the sensors tested in [58] demonstrated an effective range from pH 1 to 11. Recently a highly sensitive device [59] based on a thermally annealed GO interaction surface was also fabricated. By investigating the sensor response as a function of the type and defect density of the graphene or graphene derivative used, a better understanding of the mechanism of detection regarding pH was achieved.

The paper-based chemiresistors in [58] were created on filter paper substrates using a vacuum filtration method. The process involved depositing a suspension with mechanically exfoliated graphene, utilizing a metal mask to define the sensor geometry, and employing a vacuum to isolate the graphene on the substrate’s surface. Six sensors were batch-manufactured on each filter paper substrate. The authors explored the fabrication process’s repeatability and the sensor characteristics based on the thickness of the graphene layer, measured by weight. They produced and tested devices with graphene weights of 0.5, 1.0, and 1.5 mg, noting that the 0.5 mg variant exhibited the highest electrical stability and fabrication repeatability. Test results indicated that the 0.5 mg graphene-based device was effective over a pH range from 1 to 11 under a 1 V potential. The device’s resistance decreased as the analyte’s pH increased, due to a higher concentration of OH^−^ groups.

The sensors reported in [59] were fabricated from SLG, FLG, and GO. The FLG sensors were then functionalized with either pyrene carboxylic acid (Py-COOH), 1-amino pyrene (Py-NH_2_), or 1-hydroxypyrene (Py-OH). The SLG utilized for one of the devices reported in [59] was purchased as a sample grown via CVD on copper foil and coated in polymethyl methacrylate (PMMA). Each SLG sample was transferred in-house to a glass microscope slide acting as the device substrate, using a typical transfer method detailed by the authors. After ensuring via XPS that the SLG contained a low concentration of defects, the pristine SLG graphene device was used to obtain a baseline sensor response. The interaction mechanism of bare SLG graphene in response to pH is understood through the electrostatic gating effect, wherein the presence of hydronium ions (H_3_O^+^) in solutions with low pH results in an n-doped graphene surface and the increased concentration of hydroxide ions (OH^−^) in solutions with high pH acts as a p-type dopant on the graphene surface. After collecting baseline response data from the pristine, low-defect SLG device, both FLG- and GO-based devices were developed and functionalized.

**Figure 3:**
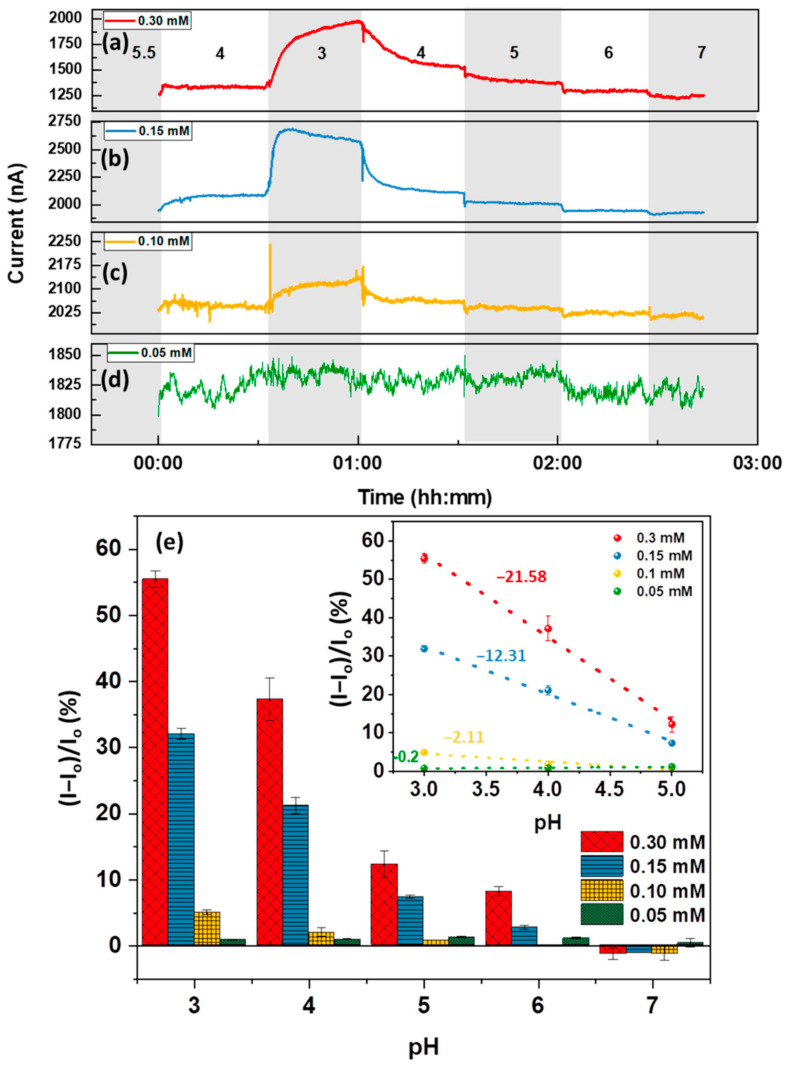
Sensing performance of 8 h annealed FLG functionalized by (**a**) 0.3 mM (Io = 1274 nA), (**b**) 0.15 mM (Io = 1980 nA), (**c**) 0.1 mM (Io = 2052 nA), and (**d**) 0.05 mM (Io = 1812 nA) of Py-COOH; (**e**) the calibration bar graph of the sensors demonstrating the highest pH response at around -COOH pK_a_ (3.1). The error bars represent the average ± standard deviation of the last two minutes of the chemiresistive response (3 samples each) [59].

FLG contains a higher concentration of surface defects than SLG, particularly when derived using a liquid exfoliation method as was the case in [59]. The increased defectivity in a sensor fabricated with FLG will influence both the sensor’s response to—and interaction with—the pH of a solution. When testing the non-functionalized FLG device, it was reported that the sensor exhibited a response that was the exact opposite of the response demonstrated by the SLG reference. This behavior was attributed by the authors [59] to a different primary method of interaction between the pH solution and the graphene surface, where instead of the electrostatic gating effect, the response is due to the protonation or deprotonation of the carboxyl, amine, and hydroxyl defect groups. After developing an understanding of how the defect groups impact the sensor response, a targeted approach to surface functionalization was possible with the objective of enhancing both the sensitivity and pH range selectivity of the response. As mentioned above, the functionalization of the FLG-based sensors was accomplished through pyrene carboxylic acid (Py-COOH), 1-amino pyrene (Py-NH2), or 1-hydroxypyrene (Py-OH), allowing the device to leverage the protonation or deprotonation interaction that was observed in the defect groups. By first annealing the FLG samples to dampen the defect-dependent response, the selectivity of the sensors could be tuned by functionalizing with the pyrene derivative that corresponds to the desired pH range. For example, Figure 3 shows the response of an FLG sensor that was functionalized using varied concentrations of Py-COOH to selectively target a pH range of 3–4.

**Figure 4:**
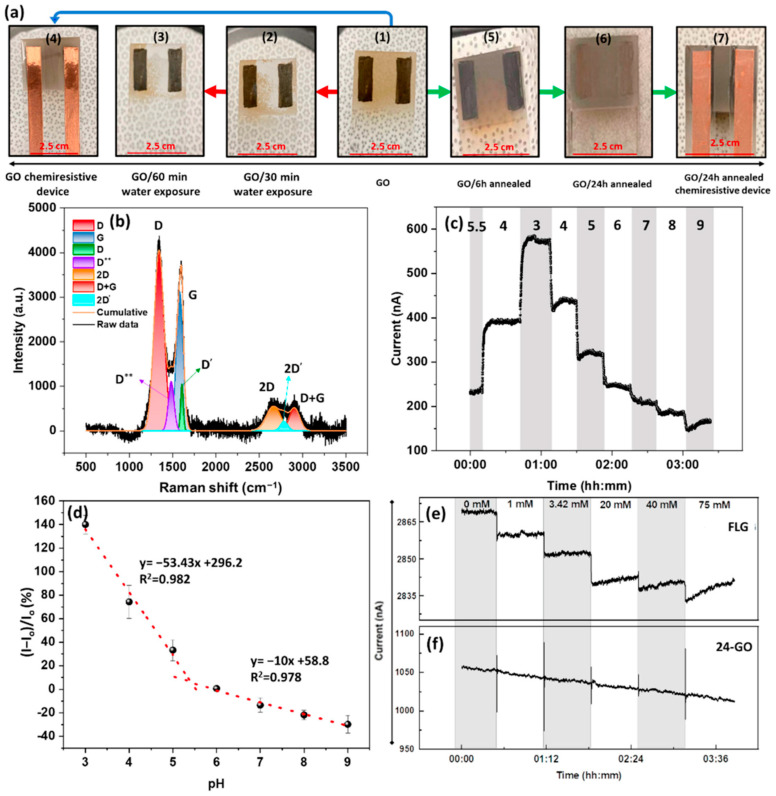
(**a**) The GO sensor fabrication: (1) Bare GO; (2) Bare GO exposed to the aqueous solution for 30 min; (3) Bare GO exposed to the aqueous solution for 6 min; (4) The GO chemiresistor without water exposure; (5) GO annealed for 6 h and (6) 26 h; (7) The 24 h annealed GO-based chemiresistor. (**b**) Deconvoluted Raman spectrum of 24 h-GO represents the presence of (from left to right): D, D**, G, D’, 2D, 2D’, D + G, and an ID/IG ratio of 1.3. (**c**) The pH response and (**d**) calibration curve of 24 h-GO (Io = 242 nA). The solution conductivity response of (**e**) FLG (Io = 2870 nA) and (**f**) 24 h-GO-based devices (Io = 1050 nA) [59].

After investigating and understanding the response of the chemiresistors based on SLG and FLG, the authors of [59] also designed, fabricated, and tested GO-based sensors. GO is a derivative of graphene that is characterized by a high defectivity density, allowing GO devices to leverage the defect-driven response examined in the pyrene-functionalized FLG samples, in a non-functionalized device. During initial testing, it was discovered that the GO layer experienced delamination when in contact with water and after only 30 min of exposure, a significant portion of the GO had been removed from the surface (Figure 4a). The Raman spectrum of GO (Figure 4b) confirms the high defectivity of the sample, showing a pronounced D peak, primarily associated with defects in the graphene oxide. To create a more robust GO surface, the sample was annealed at a low temperature of 350 °C for 24 h in a reducing atmosphere to ensure the GO retained a high number of defects. Upon testing, the GO-based chemiresistor samples exhibited a stepwise response as the pH value of the test solution was varied (Figure 4c). Notably, the GO sensor with pH sensitivity from pH 3–5 was reported as −53%/pH (Figure 4d–f); the FLG sample with an optimal surface functionalization of Py-COOH at 0.30 nM reported a sensitivity of −21.58%/pH (Figure 4e inset).

### 2.2. Disinfectant Detection

Graphene-based chemiresistive sensors are also being actively developed for the detection of chemicals classified as disinfectants by the EPA. These chemicals, which include chlorine and chlorine dioxide, are commonly introduced into drinking water supplies by water treatment facilities to deactivate harmful microorganisms like bacteria, viruses, and parasites [82]. Although ostensibly added to improve water quality, many of these chemicals can become toxic at high concentrations as well as introduce toxic DBPs, such as trihalomethanes, which have been correlated with an increased risk of cancer [83]. Table 4 presents a sample of chemicals commonly used as water disinfectants and their associated health risks, while key performance metrics of the sensors reviewed for disinfectant detection can be found in Table 5.

**Figure 5:**
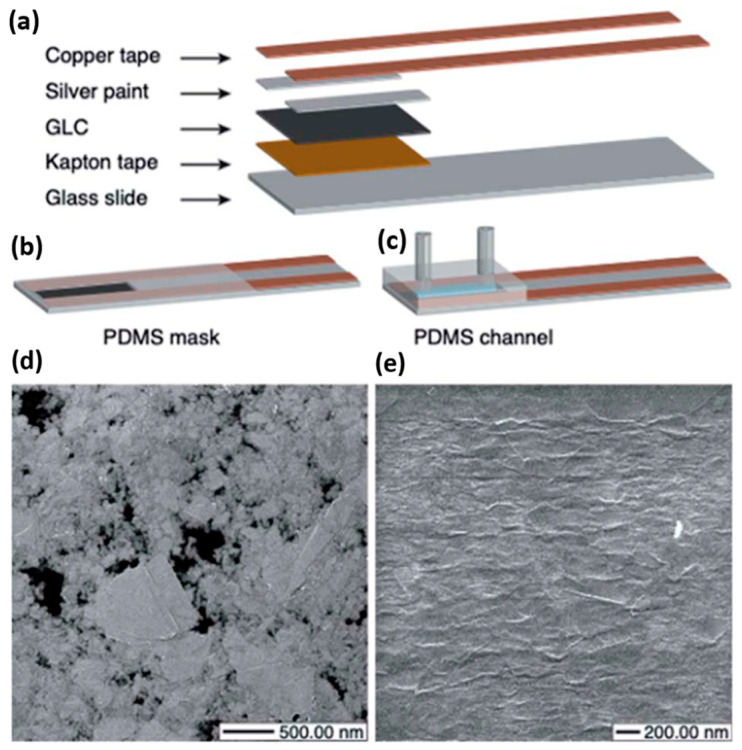
(**a**) Basic components of the chemiresistive sensors. (**b**) Dip sensor geometry. (**c**) Flow sensor geometry. (**d**) Scanning Helium Ion Microscopy (HIM) image of the 12 nm GLC sheet (×32,657.14 magnification). The black areas are exposed PET substrate. (**e**) HIM image of the 24 nm GLC sheet (×38,000.00 magnification) [60].

Chemiresistors fabricated to sense chemicals commonly used as disinfectants [60,61,62] have been reported for the detection of free chlorine in water samples. Both devices reported in [60,61] utilized phenyl-capped aniline tetramer (PCAT) to functionalize the interaction surface. PCAT is readily oxidized by chlorine and while in its oxidized state, the interaction surface experiences an electrostatic gating effect that impacts the doping concentrations and thus the electrical characteristics of the device. However, when the PCAT is in its reduced state, no doping effect is observed [84]. Both PCAT-functionalized sensors [60,61] also employed a “graphene-like carbon” (GLC) material as the sensing interface (Figure 5). The GLC film was constructed from polyethylene terephthalate (PET) sheets coated with FLG.

**Figure 6:**
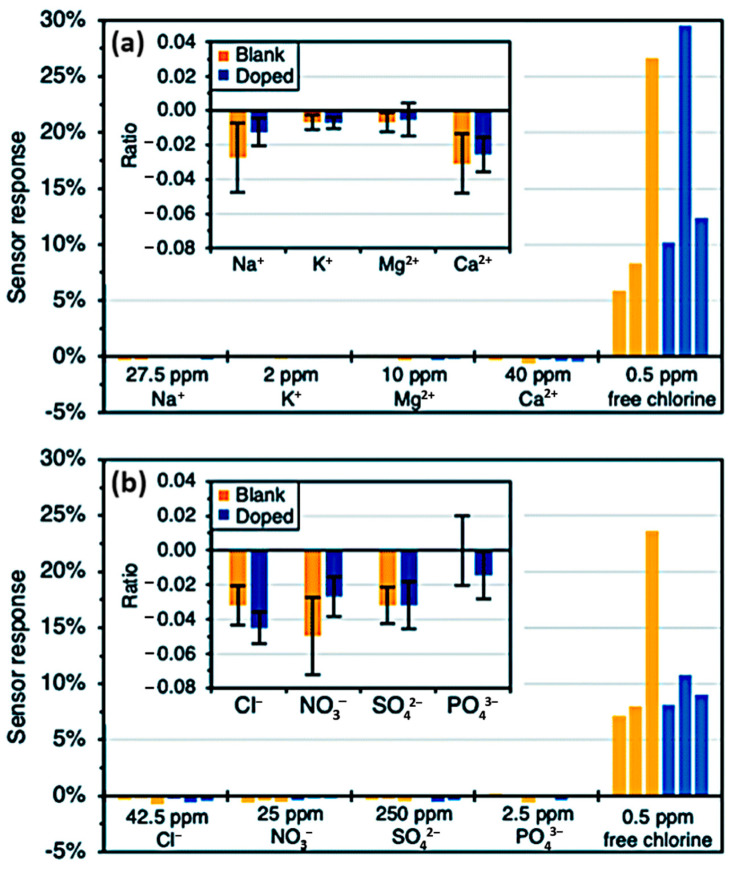
(**a**) Cationic interference study. (**b**) Anionic interference study. Insets show the average responses to the interferents in proportion to the free chlorine response (all measurements performed with 24 nm GLC at 10 mV) [60].

The chemiresistor [60] was demonstrated to have an effective working range of 0.01–1.4 ppm and a limit of detection of 1 ppb while also exhibiting high selectivity when tested in water samples containing other ions commonly found in drinking water, such as Na^+^, K^+^, NO_3_^−^, with results shown in Figure 6. This work also investigated the impact of GLC layer thickness on device sensitivity. GLC thicknesses of 12 nm, 24 nm, 38 nm, and 46 nm were used in this study. It was found that due to the primary method of interaction being the electrostatic gating effect, devices with a thicker GLC layer could support a wider range of analyte concentrations before the response became saturated; devices with a thinner GLC layer demonstrated a higher sensitivity and a lower limit of detection of 1.0 ppb [60].

The device reported in [61] also used PCAT and GLC but differed in their substrate selection, choosing to use Kapton tape in place of a glass slide to create a flexible device. During fabrication, the Kapton tape was cut to size and then copper tape electrical contacts and the GLC sensor surface were placed down. Next, the gold leaf was used to connect the contacts and GLC surface, allowing the sensor to remain flexible while improving conductivity. Finally, the gold leaf was passivated using parafilm to prevent any direct interaction between the contacts and the surrounding environment.

The flexible sensor reported an effective detection range of 0.05–1.75 ppm while withstanding repeated stress from bending up to 120°. To ensure robustness in the sensor performance after bending, three sensors underwent 15 bending cycles to 120°, and then were tested in solutions of free chlorine concentrations of 0.05, 0.16, and 0.34 ppm. The results from the stress test found no statistically significant reduction in sensing ability. Additionally, the selectivity of the chemiresistor against typical interfering compounds (sulfate, nitrate, and chloride) was tested with the concentration of interfering anions significantly higher at 500 ppm for sulfates and chlorides, and 50 ppm for nitrates, compared to the 0.34 ppm concentration of the free chlorine solution. The average sensor current response to each interferent was approximately −1.0%, where the 0.34 ppm free chlorine solution resulted in a 23.4% change in current. The device reported in [61] also demonstrated the ability to be reset through the reduction of the PCAT via ascorbic acid.

**Figure 7:**
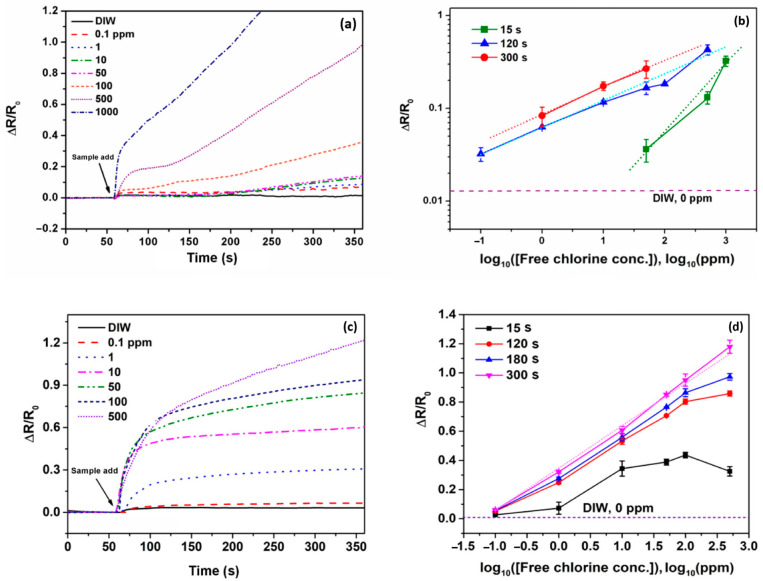
(**a**) Signal responses of PEDOT:PSS-modified paper-based sensors in solutions with different concentrations of free chlorine; (**b**) analysis of the linear relationship between the signal response of the sensor (PEDOT:PSS-modified) at different times and the logarithmic concentration of free chlorine ions; (**c**) signal response of the nanohybrid ink-modified paper-based sensors in solutions with different concentrations of free chlorine; and (**d**) analysis of the linear relationship between the signal response of the sensor (nanohybrid ink) at different times and the logarithmic concentration of free chlorine ions [62].

Lastly, a graphene-based chemiresistor [62] fabricated on a paper substrate was also reported for the detection of free chlorine by direct interaction between the analyte and graphene. This work compared the response of a chemiresistor using poly(3,4-ethylenedioxythiophene):poly(styrenesulfonate) (PEDOT:PSS) as the sensing surface with that of an identical chemiresistor employing a nanohybrid conductive ink. This ink was a composite of poly(3,4-ethylenedioxythiophene):poly(styrenesulfonate) (PEDOT:PSS) solution and graphene ink, mixed in a 7:3 ratio, used as the sensing surface. The reported results from each type of sensor, shown in Figure 7, show that the device with the graphene-enhanced ink is more sensitive, detecting free chlorine concentrations with an effective linear detection range of 0.1–500 ppm and the limit of detection of 0.18 ppm. Additionally, the chemiresistor was also integrated into a system that transmitted the sensor output to a mobile device via Bluetooth, enabling real-time monitoring of the device output.

### 2.3. Heavy Metal Detection

Another group of contaminants found in drinking water is classified as heavy metals. Some examples of metals that make up this group and are commonly found in water are lead, cadmium, mercury, chromium, and arsenic; all of which are highly toxic to humans [85]. These pollutants can be introduced to a body of water through a variety of means such as erosion, industrial pollution, or leeching from the material used in water service lines [85,86]. The presence of heavy metals in drinking water poses a serious health risk, particularly through bioaccumulation of the contaminants [87]. Due to the acute and chronic toxicities of most heavy metal contaminants, some of which are highlighted in Table 6, the EPA places strict regulations on allowable quantities in the water supply [13].

Seven graphene-based chemiresistors, as reported in references [63,64,65,66,67,68,69] were developed to detect different metal ions, including silver (Ag^+^), lead (Pb^2+^), cadmium (Cd(II)), mercury (Hg^2+^), and chromium (Cr(VI)). Key performance metrics of the sensors reviewed for heavy metal detection can be found in Table 7.

**Figure 8:**
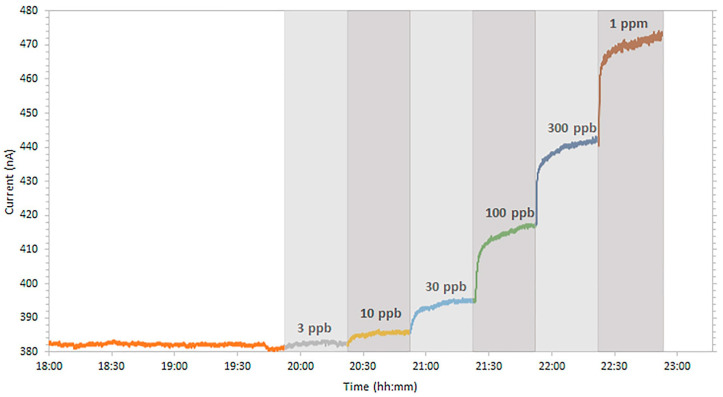
Current response to different concentrations of silver (I) in solution. Concentrations below 30 ppb took ~15 min to reach a stable current value. Above this, stabilization only took ~5 min [63].

The chemiresistor designed for the detection of silver ions [63] utilized an FLG film functionalized with bathocuproine as the sensing interface between electrical contacts. The use of bathocuproine as a selective complexing agent enabled the sensor to bind with silver ions present in the sample, thereby modifying the electrical properties of the chemiresistor. The construction of the sensor is similar to other works, using a glass slide as the substrate, followed by pencil-drawn graphite contacts, an FLG sensing layer, and copper tape contacts passivated with polydimethylsiloxane (PDMS). This sensor reported an effective linear detection range of 0.030–1 ppm, with a calculated LoD of 3.0 ppb (Figure 8). Additionally, the chemiresistor exhibited good selectivity when introduced to samples containing other species of ions, with a noted exception of copper ions (Cu^2+^), which was attributed to the chemical similarity between copper and silver. Additionally, the sensor [63] also demonstrated the ability to be reset and reused without diminished performance.

**Figure 9:**
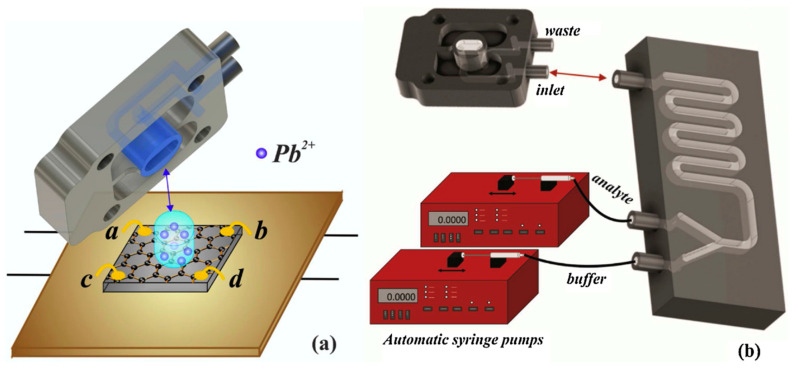
Schematic of board mounting process (**a**) and experimental setup (**b**) [64].

**Figure 10:**
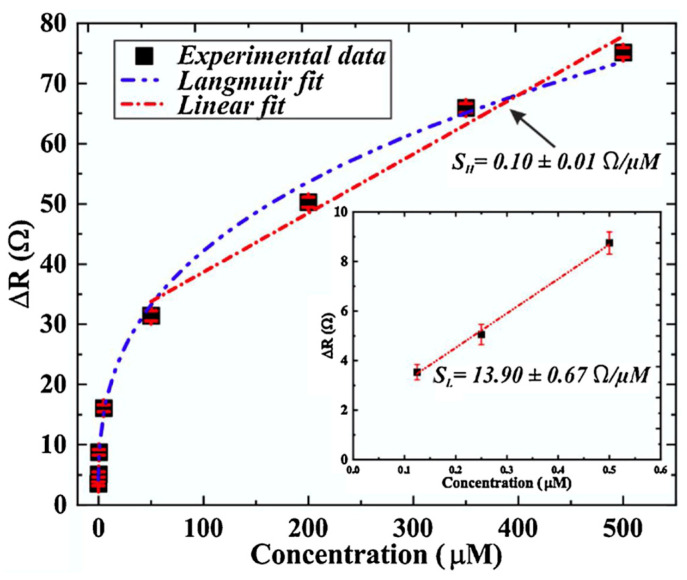
Calibration curve showing the dependence of the differential resistance on the Pb concentration. The inset zooms into the initial part of the curve at low Pb concentrations. The red straight lines are linear fits to different parts of the curve, illustrating the different sensitivity at low and high concentrations [64].

The toxicity of lead is well-known. The unfortunate pervasiveness of lead in old water networks makes its monitoring particularly critical. A graphene chemiresistor [64] designed to detect the presence of lead ions was fabricated by leveraging a microfluidic apparatus, as shown in Figure 9, to route the aqueous testing sample over the monolayer graphene sensing surface grown on a 4H-SiC substrate. Using an automated pump, the test solutions of varying lead ion concentrations were circulated over the device surface at a rate of 19.2 mL/h. The chemiresistor was tested with solutions containing lead ions at concentrations of 0.125, 0.25, 0.5, 5, 50, 200, 350, and 500 μM and was reported to be effective at detecting the presence of the analyte at all tested concentrations with a calculated LoD of 95 nM, as shown in Figure 10.

**Figure 11:**
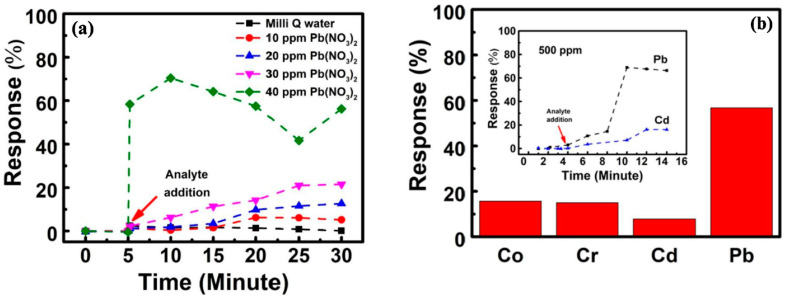
(**a**) % response shown by rGO-BCD film for Milli Q water and 10 ppm, 20 ppm, 30 ppm, and 40 ppm of the Pb(NO3)2 solution, respectively, and (**b**) comparative % response of rGO-BCD film for different heavy metal nitrate 50 ppm aqueous solutions namely cobalt (Co), chromium (Cr), cadmium (Cd), and lead (Pb). The inset shows the % response for 500 ppm nitrate salt solution of Pb and Cd on rGO-BCD film [65].

Another chemiresistive sensor for the detection of lead was fabricated and tested in [65]. This design featured a β-cyclodextrin (BCD) functionalized rGO film as the sensing interface between two silver electrical contacts. More specifically, GO was synthesized and deposited as a thin film on a cleaned glass cover slip. The film was reduced with hydrazine monohydrate and annealed at 250 °C for two hours. The BCD solution was drop-cast onto the rGO film, followed by drying and thermal treatment at 100 °C for an hour. Silver contacts were added and thermally treated at 120 °C for 10 min. The sensors were stored under vacuum for 12 h before use, labeled based on the GO:BCD weight ratio (2:1, 5:1, 10:1, 50:1, and 100:1). Testing results are shown in Figure 11. It was found that the ratio of GO to BCD was a critical factor in overall device performance. BCD molecules attached to the rGO surface via interactions with oxygen-containing functional groups like -OH and -COOH. Unlike rGO, BCD is non-conductive, which leads to increased resistivity as more BCD is mixed with rGO. Samples with weight-to-weight ratios of 2:1 and 5:1 showed high resistivity compared to ratios of 10:1, 50:1, and 100:1. Results from testing the 100:1 GO:BCD ratio device [65] variation demonstrated its capability to detect lead ions in concentrations ranging from 10 ppm to 500 ppm, with an average settling time of approximately 30 min. Notably, the device’s resistivity changed by approximately 6% at the lowest concentration (10 ppm), while concentrations at or above 40 ppm yielded response values in the range of 50–60%. The difference in measured response at analyte concentrations above 50 ppm was negligible, indicating that the sensor had entered its saturation regime. Additionally, the device exhibited selectivity for lead when tested against cobalt, chromium, and cadmium. This selectivity toward lead ions can be attributed to the hydrated diameter of the metal ion, with BCD displaying a pronounced affinity for larger ions due to its internal cavity dimensions.

**Figure 12:**
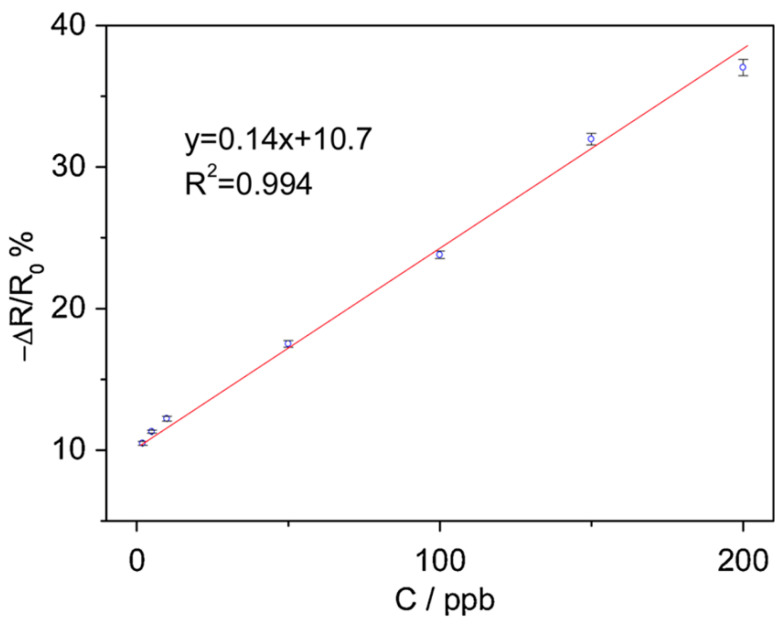
Response of IIP/rGO-CR sensor as a function of Cd(II) concentration at pH 7.0. The error bar represents ±1 standard deviation from 3 successive measurements [66].

In order to detect Cd(II) ions in water, a chemiresistor [66] was developed using an ion-imprinted polymer (IIP)-functionalized rGO sensing surface. The sensor was designed and fabricated with interdigitated electrical contacts, followed by cleaning and self-assembly of GO onto the contacts. The GO is then thermally reduced to rGO whose surface is modified with an imprinted polymer (IIP) through reversible addition-fragmentation chain transfer (RAFT) polymerization. The IIP is prepared by introducing polyethylenimine (PEI) and methacrylic acid (MAA) monomers that form a complex with target ions (cadmium ions in this case). The resulting sensor undergoes several treatments to graft the IIP onto the rGO surface. The performance of the sensor [66] was evaluated in terms of sensitivity, limit of detection, selectivity, and stability. The resistance change of the sensor exhibited good linearity with Cd(II) concentration, showing a sensitivity of 0.14 ppb (Figure 12) and a calculated limit of detection of 0.83 ppb. The selectivity of the sensor was tested by subjecting the sensor to other common heavy metal ions at a significantly higher concentration of 1000 ppb and reported a worst-case deviation from baseline of 7.1%. The long-term stability of the sensor was also investigated, revealing a change in response of 15% over a 60-day period.

**Figure 13:**
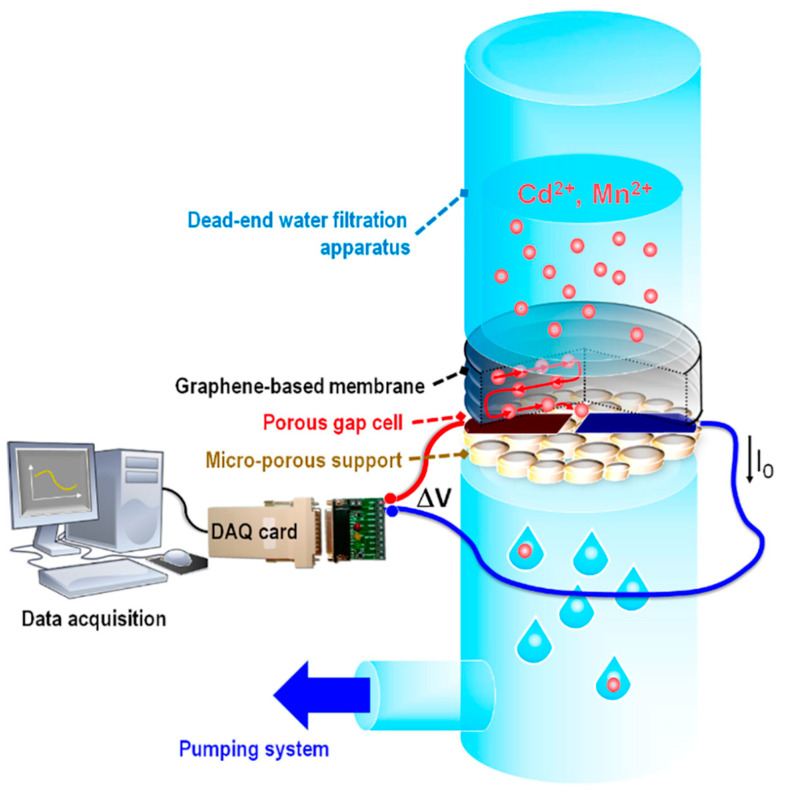
Graphene-based water filtration and sensing apparatus used in this study. The apparatus utilizes a data acquisition card to create IV curves, which allows a computer to calculate the resistivity across the gap cell. The gap cell is the area between the two aluminum contacts (black and blue). Water is filtered through this process with various types of contaminants and with varying concentrations. The graphene-based filter cake captures contaminants, which results in a change in the gap cells resistivity acquired over time by the data acquisition system [67].

Another chemiresistor-based approach to detecting cadmium in water [67] utilized an in-line nanofiltration approach. The device was fabricated using multilayer graphene cakes created through ultrasonic exfoliation in a solution of water and poly(ethylene glycol)-block-poly(propylene glycol)-block-poly(ethylene glycol) (PEG-PPG-PEG) triblock copolymer. The graphene “cake” was then created by vacuum filtration of the graphene suspension, which created a thick and stable filter medium on the poly(ether)sulfone (PES) membrane. Finally, aluminum contacts were thermally evaporated on the PES substrate to create the electrical contacts. The sensing performance of two variations of the in-line device [67] was assessed, with the water filtration and sensing apparatus used in this study shown in Figure 13. One device utilized graphene layers obtained from synthetic turbostratic graphite, while the other incorporated graphene layers derived from natural hydrothermal graphite. The sensor exhibited a response due to a change in resistivity to Cd^2+^ ions, detecting concentrations between 5 and 125 ppb in a linear regime and up to 500 ppb in a saturation regime. Notably, the sensor exhibited selectivity toward Cd^2+^ ions when compared to manganese and mercury ions. This selectivity was attributed to the mechanism of interaction between the sensor and the analyte, specifically the physical entrapment of Cd^2+^ ions within the multilayer graphene when their hydrodynamic diameter closely matched the interlayer spacing between graphene layers. In terms of material performance, natural graphite outperformed its synthetic counterpart.

**Figure 14:**
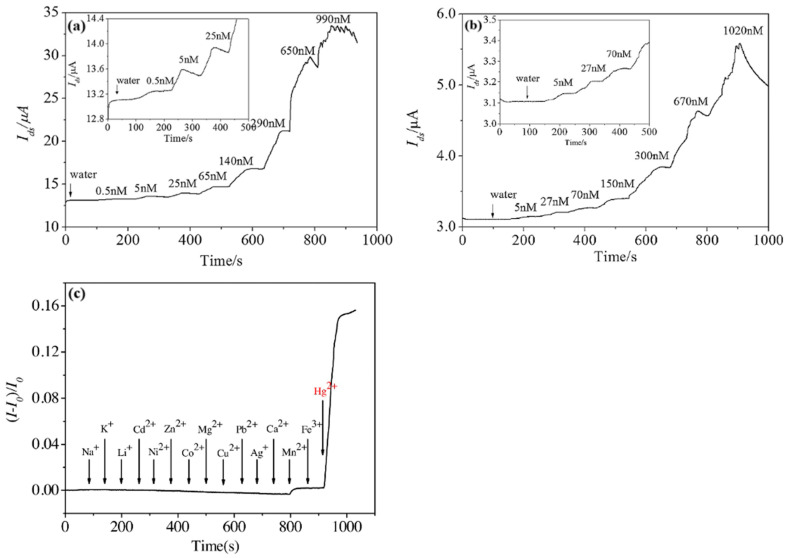
Real-time response of the electrically derived rGO chemiresistor (**a**) nd chemically derived rGO chemiresistor (**b**) biosensors upon exposure to different concentrations of Hg^2+^ ion (Vds = 0.1 V). The concentrations shown in the figures are the cumulative concentrations after the sequential addition of the Hg^2+^ ion. (**c**) Responses of the electrically derived rGO chemiresistor (V_ds_ = 0.1 V) to Hg^2+^ ion (100 nM) and other metal ions including Na^+^, K^+^, Li^+^, Cd^2+^, Ni^2+^, Zn^2+^, Co^2+^, Mg^2+^, Cu^2+^, Pb^2+^, Ag^+^, Ca^2+^, Mn^2+^, and Fe^3+^ ions (final concentrations: 2.9 μM 4.8 μM) [68].

A graphene chemiresistor for the detection of mercury was also reported [68]. This sensor features an interdigitated electrical contact design and the use of 1-pyrenebutanoic acid succinimidyl ester (PASE) and a single-stranded DNA aptamer to functionalize the rGO sensing material. The specific DNA aptamer (5′-(NH2)TTCTTTCTTCCCCTTGTTTGT-3′) was deliberately chosen for its high affinity for binding with Hg^2+^ ions. The results from testing, as shown in Figure 14, show a response to concentrations of Hg^+^ ions down to 0.5 nM. To assess selectivity, the sensor [68] was exposed to a solution containing Na^+^, K^+^, Li^+^, Cd^2+^, Ni^2+^, Zn^2+^, Co^2+^, Mg^2+^, Cu^2+^, Pb^2+^, Ag^+^, Ca^2+^, Mn^2+^, and Fe^3+^ ions, which were sequentially added over a period of time. As shown in Figure 14c, there is a notable response in the form of a ~0.5% increase in current upon the introduction of the Mn^2+^ ions, however, when the target analyte (Hg^2+^) ion is introduced, a much larger response of ~15.1% is observed.

**Figure 15:**
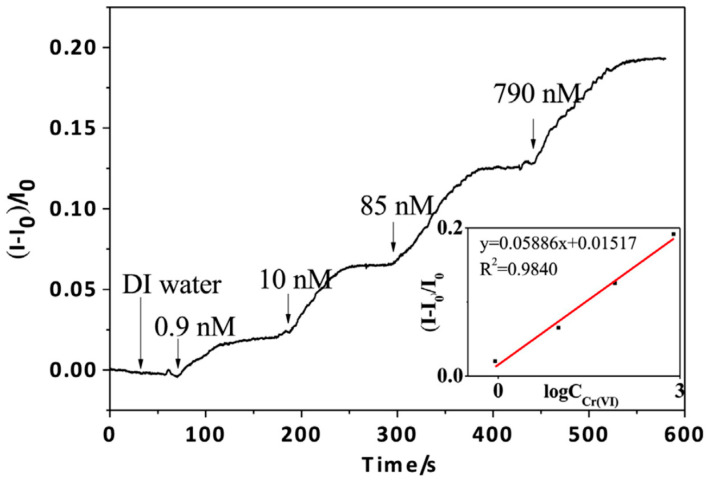
Real-time response of the sensor upon exposure to different Cr(VI) concentrations at a fixed voltage (Vds = 0.1 V). The concentrations in the figure are the cumulative concentrations after the sequential addition of Cr(VI) [69].

Another chemiresistor sensor [69] for the detection of Cr(VI) was fabricated on an Si/SiO_2_ wafer with Au-interdigitated electrical contacts. A GO suspension was cast onto the contacts, followed by an electrochemical reduction to rGO using a three-electrode system, and finally annealed at 150 °C for 1 h under an ambient atmosphere. Additionally, two types of gold nanoparticles, AuNPs1 and AuNPs2, were prepared with diameters of ~6 nm and ~20 nm, respectively. The interdigitated contacts at the sensing surface were passivated using methoxy PEG thiol (mPEG-SH) to prevent their direct interaction with the target analyte. AuNPs1 were then adsorbed onto the rGO conductive channel through electrostatic interaction, and the resulting structure was incubated in a 1,4-dithiothreitol (DTT) solution to obtain rGO with the DTT-AuNPs1 channel. The detection strategy involves selective binding between DTT-AuNPs1 in the rGO channel and DTT-AuNPs2 in solution, forming disulfide bonds. Simultaneous exposure to DTT-AuNPs2 and Cr(VI) leads to aggregation in the rGO channel, which increases conductivity. The response is observed with the presence of Cr(VI) but not with DTT-AuNPs2 alone. When tested, [69] reported a detection range of 0.9–800 nM, as shown in Figure 15. Additionally, the sensor demonstrated selectivity in the presence of Na^+^, K^+^, Ca^2+^, Ni^2+^, Zn^2+^, and Pb^2+^ ions in the water sample, however, a significant response was noted in the presence of Al^3+^, Fe^3+^, Mn^2+^, and Hg^2+^ ions.

### 2.4. Volatile Organic Compound Detection

Volatile organic compounds (VOCs) are a group of organic chemicals that have a high vapor pressure at room temperature and are often used in industrial processes, consumer products, and as fuel. They can be found in various forms, such as natural gas, oil, and gasoline, and may contaminate drinking water sources if not properly handled and stored. Exposure to VOCs in drinking water can pose serious health risks, including damage to the liver, kidneys, and central nervous system [88]. Table 8 presents a sample of VOCs, along with their health risks and common sources of pollution. To minimize the potential health hazards, regulatory agencies such as the EPA impose strict regulations on the allowable quantities of VOCs in drinking water. Therefore, monitoring and detection of VOCs in drinking water is necessary to ensure safe and healthy water sources for human consumption. Key performance metrics of the sensors reviewed for VOC detection can be found in Table 9.

**Figure 16:**
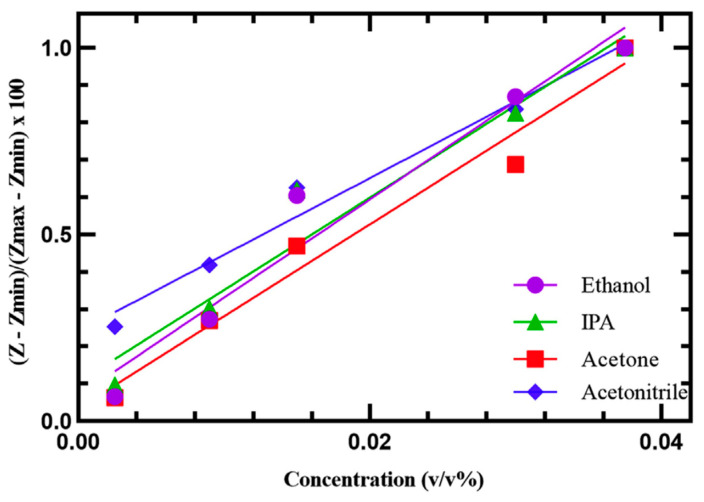
The calibration curve for ethanol (R^2^ = 0.93), IPA (R^2^ = 0.95), acetone (R^2^ = 0.98), and acetonitrile (R^2^ = 0.98) by graphene chemiresistive sensors [70].

Two graphene chemiresistive sensors [70,71] were reported to detect the presence of VOCs in aqueous solutions. The graphene sensor in [70] was developed to target ethanol, isopropanol, acetone, and acetonitrile dissolved into liquid media. The sensor fabrication process involved cleaning the glass substrate, depositing a layer of chromium (Cr) and gold (Au) with a thickness of 25 nm and 75 nm, respectively, and etching out the excess metal to create interdigitated electrical contacts. The sensing film was a composite film of graphene powder and polymethyl methacrylate (PMMA). Figure 16 displays the reported test results for ethanol, IPA, acetone, and acetonitrile that were obtained under optimal conditions, including an applied voltage of 100 mV and a frequency range of 100 kHz to 1 Hz with 50 steps/dec. The sensor exhibited a linear response to all four target analytes within a concentration range of 1.96–69 ppt.

**Figure 17:**
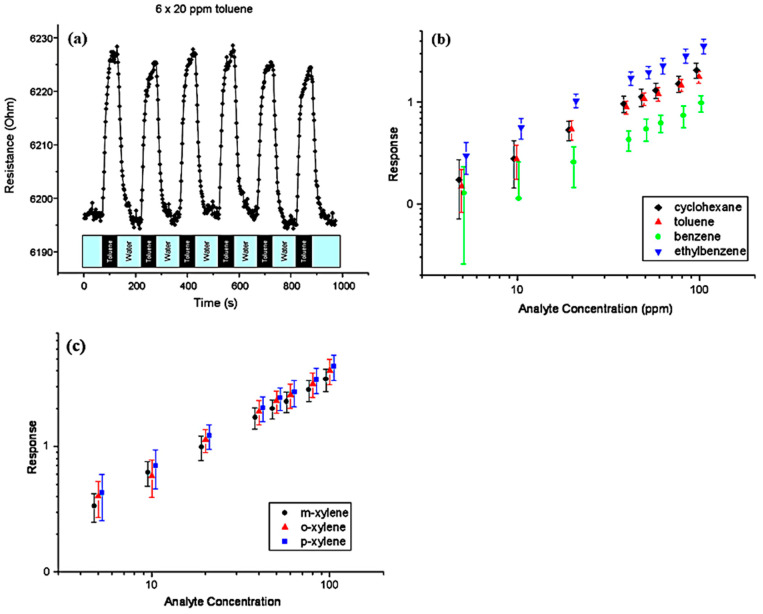
(**a**) Time vs. resistance profile of an ODA functionalized graphene sheet chemiresistor to an on/off response of a 20 ppm solution of toluene. (**b**) Equilibrium responses of ODA functionalized graphene chemiresistors to cyclohexane, toluene, benzene, and ethylbenzene dissolved in water at various concentrations (5–100 ppm). The different datasets have been offset in the x-axis by 3% for clarity. (**c**) Equilibrium responses of ODA functionalized graphene chemiresistors to m-xylene, o-xylene, and p-xylene dissolved in water at various concentrations (5–100 ppm). The different datasets have been offset in the x-axis by 5% for clarity [71].

The second graphene sensor device targeting VOCs [71] focused on the detection of benzene, toluene, ethylbenzene, xylenes (collectively referred to as BTEX), and cyclohexane. This chemiresistor design also featured gold interdigitated electrical contacts and utilized a drop-casting method to disperse the rGO. To functionalize the sensing surface, octadecylamine was used. During testing, the device was exposed to benzene, toluene, ethylbenzene, cyclohexane, and xylene isomers at concentrations ranging from 5 to 100 ppm while the contacts were biased at 100 mV and the resistance was continuously measured. The sensor exhibited rapid response with a settling time of less than 1 min for toluene detection as well as the ability to be quickly reset within 1 min by using water, as shown in Figure 17a. In addition to the rapid response, the sensor also demonstrated a linear response to all of the analytes throughout the tested concentration range, as shown in Figure 17b,c.

## 3. Future Development

Graphene-based chemiresistors represent an emerging technology with significant potential in water quality monitoring. These sensors offer solutions to many of the problems that currently plague the industry, including high costs, limited coverage, and low throughput. While undeniable progress has been made, there is still much work to be done before the commercialization and widespread adoption of these devices can become a reality. If these sensors are going to be seriously considered for use in industry, they must be cost-competitive. While the architecture of the chemiresistor is simple and low cost to manufacture, the production of high-quality graphene is still an early-stage industry. However, significant strides have been made in recent years [89], and the industry has experienced rapid growth [90,91]. One benefit of graphene is that due to its remarkable material properties, it is highly desired in many different applications and across various industries [92]. This high utility will naturally lead to increased demand, driving continuous advancements in production. Regarding the development of first-of-a-kind graphene-based chemiresistive sensors, a clear starting point is to design devices to detect target analytes that have yet to be explored within the domain of water quality monitoring. As mentioned earlier, the EPA currently has regulations in place for eighty-nine unique drinking water contaminants. However, this review only found graphene chemiresistor sensors for sixteen different drinking water analytes at the time of writing. This represents a significant research opportunity that can be easily leveraged while also serving the critical purpose of expanding the range of applications for graphene-based chemiresistors. Once devices have been validated for a wide range of individual analytes, the natural next step would be to enhance the utility of each water sample by creating a sensor array capable of testing multiple analytes simultaneously. Another approach is to improve one or more of the three primary traits: sensitivity, selectivity, and response time for a given sensor/analyte pair. To accomplish this, graphene’s high sensitivity to the outside environment needs careful consideration to ensure the target analyte is the primary modulator of the output signal from the device. To address this, one could potentially leverage graphene’s strong affinity for binding to other aromatics due to the π-π interactions to develop novel surface functionalization schemes. Performance improvements in these areas can first be made in a controlled lab environment as a proof-of-concept but ultimately must be able to perform when exposed to a real-world sample. Of the surveyed devices above [61,65,67,68] were tested under a simulated real environment by exposing the device to samples containing additional species mixed with the target analyte. The devices reported in [60,62,63,64,66,69] took this testing one step further and exposed the device to an actual sample collected from a real-world water source. The remaining devices [56,57,58,59,70,71] were tested only under laboratory conditions and with samples containing only the target analyte. It is crucial to ensure that the devices can detect target analytes at concentrations low enough to meet enforced limits, are selective enough to detect the analyte in real-world water samples, and can respond within a reasonable timeframe. Once these performance metrics are achieved for a given device, attention can be directed toward improving other aspects, such as lifespan, reset and reuse capabilities, and low-cost manufacturing techniques.

## 4. Conclusions

WQM is a critical part of water infrastructure and is essential for ensuring safe drinking water for communities. The development of graphene-based chemiresistive sensors to replace or augment existing industry-standard methods for WQM is a promising area of research with the potential for real-world application. The development of these sensors has progressed rapidly in recent years, with new designs, functionalization methods, and detection mechanisms. Despite the remaining challenges, such as expanding the number of analytes successfully sensed and improving the three primary FoM (sensitivity, selectivity, and response time) for a given sensor/analyte pairs to meet or exceed current industry levels, graphene-based chemiresistors have the potential to revolutionize WQM by providing highly accurate and cost-effective sensors. This concise review has provided a comprehensive summary of recent developments in graphene-based chemiresistive sensors, highlighting the promising future of this technology in addressing the critical need for WQM. Overall, further research is needed to address the remaining challenges and optimize the performance of these sensors for practical applications in real-world applications.

## Figures and Tables

**Table 1:** EPA table of National Primary Drinking Water Regulations [13].

Contaminant	Potential Health Effects	Common Sources of Contaminants in Drinking Water
Disinfectants		
Chlorine (as Cl_2_)	Eye/nose irritation; stomach discomfort	Water additive used to control microbes
Chlorine dioxide (as ClO_2_)	Anemia; infants, young children, and fetuses of pregnant women: nervous system effects	Water additive used to control microbes
Heavy Metals		
Copper	Short-term exposure: gastrointestinal distress. Long-term exposure: Liver or kidney damage. People with Wilson’s disease should consult their doctor if the amount of copper in their water exceeds the action level.	Corrosion of household plumbing systems; erosion of natural deposits
Lead	Infants and children: Delays in physical or mental development; children could show slight deficits in attention span and learning abilities; adults: kidney problems; high blood pressure	Corrosion of household plumbing systems; erosion of natural deposits
VOCs		
Toluene	Nervous system, kidney, or liver problems	Discharge from petroleum factories
Vinyl Chloride	Increased risk of cancer	Leaching from PVC pipes; discharge from plastic factories
Xylenes (total)	Nervous system damage	Discharge from petroleum factories; discharge from chemical factories

**Table 2:** Graphene-based water quality chemiresistive sensors.

Analyte	Method of Functionalization	Transducing Film	Substrate	Contact Material	LoD	Year Published	Reference
pH							
pH	Direct interaction	Graphene	Si/SiO_2_ (285 nm)	Pt/Ag	pH: 4–10	2011	[56]
pH	Direct interaction	SLG + FLG	Si/SiO_2_ (200 nm)	Cr/Au	pH: 5–9	2012	[57]
pH	Direct interaction	MLG	Paper	Graphene	pH: 1–11	2016	[58]
pH	Direct Interaction, pyrene carboxylic acid (Py-COOH), 1-amino pyrene (Py-NH_2_), and 1-hydroxypyrene (Py-OH)	SLG, FLG, GO	Glass	Graphite/Cu	pH: 3–9	2022	[59]
Disinfectants							
Free Chlorine	phenyl-capped aniline tetramer (PCAT)	Graphene-like carbon (GLC)	Glass	Ag/Cu	0.001 ppm	2022	[60]
Free Chlorine	phenyl-capped aniline tetramer (PCAT)	Graphene-like carbon (GLC)	Kapton Tape	Au (24 Karat Gold-leaf)/Ag	NR	2021	[61]
Free Chlorine	Direct interaction	PEDOT:PSS + Graphene composite	Paper	Ag	0.18 ppm	2020	[62]
Heavy Metals							
Ag^+^	Bathocuproine	FLG	Glass	Graphite/Cu	0.003 ppm	2021	[63]
Pb^2+^	Direct interaction	SLG	4H-SiC	Ti/Au	0.02 ppm	2019	[64]
Pb^2+^	β-cyclodextrin	rGO	Glass	Ag	10 ppm	2021	[65]
Cd^2+^	Ion-imprinted polymer	rGO	Si/SiO_2_ (300 nm)	Au	0.00083 ppm	2021	[66]
Cd^2+^	Poly(ethylene glycol)-poly(propylene glycol)-poly(ethylene glycol) (PEG-PPG-PEG) block copolymers	MLG	poly(ether)sulfone (PES)	Al	NR	2019	[67]
Hg^2+^	Single-stranded DNA aptamer	rGO	Si/SiO_2_ (300 nm)	Au	NR	2016	[68]
Cr(VI)	DTT-AuNPs	rGO	Si/SiO_2_ (300 nm)	Au	0.00047 ppm	2017	[69]
VOCs							
ethanol, isopropanol, acetone, and acetonitrile	Direct interaction	PMMA + Graphene nanopowder	Glass	Cr/Au	NR	2020	[70]
benzene, toluene, ethylbenzene, xylenes and cyclohexane	Octadecylamine	rGO	Glass	Au	Benzene: 10 ppm Cyclohexane: 5 ppm Ethylbenzene: 3 ppm Toluene: 5 ppm Xylenes (all): 3 ppm	2011	[71]

NR = Not Reported.

**Table 3:** Device performance for pH detection.

Analyte	Linear Detection Range	Response Time [min] *	EPA Regulatory Limit **	Device
pH	4–10 pH	~2	6.5–8.5	[56]
pH	5–9 pH	~1	6.5–8.5	[57]
pH	1–11 pH	~1	6.5–8.5	[58]
pH	3–9 pH	~10	6.5–8.5	[59]

* Response time is approximate, where not explicitly stated in the reference. ** EPA recommended range for pH.

**Table 4:** Health hazards associated with disinfectants. Adapted from [13].

Disinfectants	Potential Health Effects	Common Sources of Contaminants in Drinking Water
Chlorine	Eye/nose irritation; stomach discomfort	Water additive used to control microbes
Chloramines	Eye/nose irritation; stomach discomfort; anemia	Water additive used to control microbes
Chlorite	Anemia; infants, young children, and fetuses of pregnant women: nervous system effects	Byproduct of drinking water disinfection

**Table 5:** Device performance for disinfectant detection.

Analyte	Linear Detection Range	Response Time [min] *	EPA Regulatory Limit [ppm]	Device
Free chlorine	0.01–1.40 ppm	30	4.00	[60]
Free chlorine	0.05–1.75 ppm	~30	4.00	[61]
Free chlorine	0.10–500 ppm	5	4.00	[62]

* Response time is approximate where not explicitly stated in reference.

**Table 6:** Health hazards associated with toxic heavy metals. Adapted from [13].

Metals	Potential Health Effects	Common Sources of Contaminants in Drinking Water
Arsenic	Skin damage or problems with circulatory systems and may have increased risk of getting cancer	Erosion of natural deposits; runoff from orchards; runoff from glass and electronics production wastes
Cadmium	Kidney damage	Corrosion of galvanized pipes; erosion of natural deposits; discharge from metal refineries; runoff from waste batteries and paints
Chromium	Allergic dermatitis	Discharge from steel and pulp mills; erosion of natural deposits
Lead	Infants and children: Delays in physical or mental development; children could show slight deficits in attention span and learning abilities; adults: kidney problems; high blood pressure	Corrosion of household plumbing systems; erosion of natural deposits
Mercury	Kidney damage	Erosion of natural deposits; discharge from refineries and factories; runoff from landfills and croplands

**Table 7:** Device performance for heavy metal detection.

Analyte	Linear Detection Range	Response Time [min] *	EPA Regulatory Limit	Device
Silver	0.03–1.00 ppm	~5	0.1 ppm	[63]
Lead	0.03–103.6 ppm	<1	0.015 ppm	[64]
Lead	10.0–50.0 ppm	30	0.015 ppm	[65]
Cadmium	2–200 ppb	NR	5 ppb	[66]
Cadmium	5–125 ppb	~1	5 ppb	[67]
Mercury	0.1–200 ppb	~<1	2 ppb	[68]
Chromium	0.05–41.6 ppb	~<1	100 ppb	[69]

* Response time is approximate where not explicitly stated in reference.

**Table 8:** Health hazards associated with toxic VOCs. Adapted from [13].

VOCs	Potential Health Effects	Common Sources of Contaminants in Drinking Water
Benzene	Anemia; decrease in blood platelets; increased risk of cancer	Discharge from factories; leaching from gas storage tanks and landfills
Ethylbenzene	Liver or kidney problems	Discharge from petroleum refineries
Toluene	Nervous system, kidney, or liver problems	Discharge from petroleum factories
Trichloroethylene	Liver problems; increased risk of cancer	Discharge from metal degreasing sites and other factories
Xylenes	Nervous system damage	Discharge from petroleum factories; discharge from chemical factories

**Table 9:** Device performance for VOC detection.

Analyte	Linear Detection Range	Response Time [min] *	EPA Regulatory Limit [ppm]	Device
Ethanol, isopropanol, acetone, and acetonitrile	1.96–69 ppt	8	NR	[70]
Benzene, toluene, ethylbenzene, xylenes, and cyclohexane	5–100 ppm	<1	benzene: 0.005; toluene: 1; ethylbenzene: 0.7; xylenes: 10; cyclohexane: NR	[71]

* Response time is approximate where not explicitly stated in reference.

## Data Availability

No new data were created or analyzed in this study. Data sharing is not applicable to this article.
